# Reconfiguration of low-voltage distributed power sources within electric power's distribution network based on improved particle swarm-fish swarm fusibility algorithm

**DOI:** 10.1038/s41598-024-56131-0

**Published:** 2024-03-05

**Authors:** Xiaowei Xu, Ding Nie, Wenhua Xu, Enxin Xiang, Shan Chen, Yongjie Nie, Xiao Fu, Wan Xu, Yiming Han

**Affiliations:** 1 Yunnan Electric Power Grid Research Institute, Kunming, 650217 China; 2 Faculty of Electric Power Engineering, Kunming University of Science and Technology, Kunming, 650504 China

**Keywords:** Distributed power supply, Distribution network reconfiguration, Particle swarm algorithm, Artificial fish swarm algorithm, Objective function, Engineering, Mathematics and computing

## Abstract

With the development of distributed power sources in the distribution network, the algorithm of distribution network reconfiguration is gaining attention from experts and scholars. Its goal is to reduce the power loss during power transmission, so as to reduce the power grid loss during power transmission. And weaken the electric heating effect in the process of electric energy transmission, thus maintaining the safety of the surrounding residents. Due to the wire impedance effect, a lot of electric energy of the circuit is lost to electric heating, which is easy to cause local overheating and lead to fire. This will not only cause power loss, but also endanger the safety of surrounding residents. To address the issue, experiments on distribution grid reconstruction are performed using the enhanced particle swarm-fish swarm algorithm with the Elecgrid self-constructed dataset. Initially, low-voltage distributed power sources in parallel are connected to the circuit, thereby decreasing internal resistance and electrical heat. Then, by controlling the circuit in the system, the double separation relay adjusts the inductance and capacitance of the conductor, thus reducing the reactance length. Additionally, particle swarm particles are mutated to enable them to jump out of the local optimum, and elite fish approach is used to expand the search area. Finally, the proposed fusion algorithm is applied to the self-built data set of Elecgrid and compared with the other three algorithms. The fusion algorithm serves as the standard test system for this comparison. The active power loss of the hybrid algorithm is 63 kW at an operating voltage of 0.74 V. The loss work of the other three algorithms is 74 kW, 97 kW and 109 kW respectively. The mixed algorithm has the lowest loss among the four algorithms. The experiments are repeated for six times, and the linear fitting degrees of the four algorithms are 0.9804, 0.9527, 0.9612 and 0.9503, respectively. The experimental results show that the application of this algorithm can effectively reduce the active loss in the process of distribution network reconfiguration, thus reducing energy consumption; At the same time, it can reduce the electric heating in the process of electric energy transmission, and then prevent the occurrence of fire. There are three main contributions of this study. Firstly, the resistance in the transmission path is reduced by using this algorithm, so that the power transmission efficiency can be analyzed more accurately. Secondly, the new algorithm enriches the power safety maintenance method; Finally, the fire caused by local overheating of the line is reduced by fusion algorithm.

## Introduction

Nowadays, household appliances have occupied the majority of residents' lives. Efficient transmission is not only related to economic savings, but also to the personal safety of residents. In the power system, the distribution network is the bridge between the power plant and the residents, and usually works in a long-distance transmission mode. Longer conductors result in higher resistance reactance, leading to increased electricity lost to electric heat during transmission^1^. During the delivery process, multiple components installed along the path affect the charge distribution^2^. Even if a branched switch is used for its disconnection operation, the consequent increase in switch maintenance costs can increase the impact. Recently, the fusion algorithm of the Artificial Fish Swarm algorithm (AF) and Particle Swarm Optimization algorithm (PSO) has garnered significant attention from experts due to its capacity for global search and its potential for studying distribution network reconfiguration^3^. However, PSO-AF is only applicable to distribution networks in medium and long distances. When the power grid distance is less than 10 km or more than 25 km, the model has the problem of slow data transmission. It is easy to become trapped in a local optimum when operating over expansive distances, resulting in the algorithm being terminated prematurely^4^. To solve this problem, this study creatively optimizes PSO-AF algorithm based on the strategy of mutant Birds (b) and Elite fish (e). The novelty of this paper lies in generating fusion algorithm (bPSO-eAF). The b can mutate the particles when the algorithm falls into a local optimum. The e is more active and has a wider search range, which can effectively target long-distance power delivery. When multiple components affect the charge distribution, the bPSO-eAF algorithm is used to reduce the inductance and capacitance of the power grid system and improve the transmission capacity and stability of the system. The study is mainly divided into four parts. The first part mainly analyzes and summarizes the current applications and effects of PSO and AF. The second part introduces the factors affecting the transmission efficiency and constructs the bPSO-eAF reconstruction model. The third part analyzes and compares the performance of this optimization model with the traditional model. The last part conducts simulation experiments on the Elecgrid dataset to propose the shortcomings that still exist in the study. There are three main contributions of this study. Firstly, the resistance in the transmission path is reduced by using this algorithm, so that the power transmission efficiency can be analyzed more accurately. Secondly, the new algorithm enriches the power safety maintenance method; Finally, the fire caused by local overheating of the line is reduced by fusion algorithm. The objective is to address the issue of significant losses in bPSO-eAF transmission over long distances. The research aims to achieve the goal of reducing the risk of fire caused by local overheating of power lines, thereby reducing the loss of fossil fuel energy.

## Related works

The reconfiguration of low-voltage distributed power sources within electric power's distribution network plays a critical role in ensuring the safety of residents and regulating energy consumption. Liao et al.^5^ designed parameter fitting methods based on particle swarm and fish swarm hybrid algorithms. They took the value of experimental variance given by the variance function as the optimization objective, and transformed it into a minimization problem by setting the objective function. Their hybrid algorithm has strong convergence and is able to achieve satisfactory fitting values. They compared the fitting results of VARFIT and the optimization algorithm. The fitting results of the optimization algorithm are lower than VARFIT by 3.39. The experimental results showed that their method has strong merit-seeking ability, high accuracy of merit-seeking, and can effectively achieve automatic fitting of parameters. Yang et al.^6^ analyzed and summarized the advantages and disadvantages based on the operational efficiency of wireless sensor networks. They also proposed a clustering method based on the K-mean algorithm. In order to maximize network coverage with guaranteed quality of service, they proposed a wireless sensor network coverage optimization method based on improved artificial fish swarm algorithm. They conducted controlled experiments to analyze the efficacy of their proposed algorithm. The experimental results showed that their method has some advantages and can provide a model reference for related types of research. Jain et al.^7^ proposed robust hybrid artificial fish swarm simulated annealing optimization algorithm to ensure network security. The robustness of wild networks against malicious hackers was improved without changing the temperature distribution. Their algorithm eliminated the immeasurable oscillations of the artificial fish swarm algorithm and also speed up the convergence. To model the generation of free-scale networks, they used real-world networks to test the results of synthetic free-scale. The experimental results showed that their model outperforms other models in several aspects.

Gunawan et al.^8^ presented four new independent optimization algorithms for solving single-objective problems in renewable energy sources. They used the IEEE 30-node test system to compare the proposed metaheuristic optimization with alternatives, including particle swarm, moth flame optimization, and gray wolf optimization. To address the challenges in modern power models, they proposed to test under different operating conditions to determine the presence or absence of renewable energy sources or renewable energy locations. The results showed that their proposed algorithm was able to solve the problem more efficiently compared to other algorithms. Their algorithm improved the deviation term by 27% compared to the PSO algorithm. Zhang et al.^9^ proposed the generalized space transformation evolution technique and combined it with an improved particle swarm optimization algorithm. Their generalized space was based on contrastive learning, which not only improves the utilization of the current space, but also enhances the exploration of the current space. The improved algorithm used spatial transformation search for generation jumps, and experiments on well-known unconstrained benchmark functions showed empirical evidence of the contribution of the generalized space. They also compared quantum-behaving particle swarm with some typical extension methods. The comparison showed that their optimization algorithm has promise compared to another algorithm. Yoganand et al.^10^ used two algorithms, genetic algorithm and particle swarm algorithm, to solve the service combination problem. Due to the large number of services with similar functions, different characteristics have become a key issue in service management. Service quality consists of different factors, such as service cost and reliability. They selected the right supply chain based on IoT to meet the user needs, which is the key problem they solve. They used particle swarm to solve the supply chain problem and further evaluation. Comparative results showed that genetic algorithm can improve the efficiency of the supply chain and outperform the results of particle swarm algorithm. Zhang^11^ believes that distributed technology has strong application performance in highway engineering, especially for expressways. He used distributed power supply in the tunnel and found its superiority. In order to realize the effective application of this technology, the expressway is selected for tunnel power supply, and its application effect is explored. The experimental results show that this technology has advantages, and it has application ability in expressway tunnels, which can provide reference for the distribution network reconstruction of expressway distributed power supply. Daus et al.^12^ considered the penetration effect of photovoltaic technology in grid connection, improved the efficiency of the optimized distributed power supply in parameter selection. According to their distributed power generation system, the solar intensity will decrease with the consumption of the network. Finally, they carried out the experiment of distributed power supply, and the experimental results show that their method is effective. Naguib et al.^13^ put forward the method of distribution network reconfiguration, which can reduce the loss of distribution network in distributed power supply and improve the working environment of distribution system. Based on renewable energy, they discussed its intermittent load curve and minimized the annual energy loss to provide energy. The key point of this method is the firefly algorithm, and the experiment is carried out on the node distribution system. The results of several cases show that this method is effective in reducing power grid loss.

Due to the complexity and variability of distribution network, there are some shortcomings in recent works, such as the uncertainty of algorithm. These methods mainly focus on theory and simulation, and have not been applied and verified on a large scale. In addition, their methods need more data support and actual case analysis to further improve and improve. Articles from various scholars indicate that there is international interest in algorithms for reconfiguring distribution networks. However, fusion algorithms have not yet received much attention. For the first time, mutant birds and elite fish were combined with PSO-AF. The fusion algorithm (bPSO-eAF) was generated considering the impact of components such as low-voltage distribution power supplies and relays.

## Modelling of distribution network reconfiguration for distributed power supply in low voltage state

With the development of the power industry, low-voltage relay protection is becoming increasingly importan. Moreover, the research on distribution network reconstruction is becoming increasingly popular. However, objective factors such as high heat loss and slow transmission in the distribution network increase the difficulty of model reconstruction. These further increase the difficulty of relay protection. This paper combines the Particle swarm optimization algorithm (PSO) with the Artificial Fish Swarm algorithm (AF). First, the models developed based on both are presented, followed by an explanation of how they are combined to achieve 35 kV and below relay protection. Particle Swarm Optimization-Fish Swarm Algorithm has good parallel processing ability and can search globally in the whole distributed power supply. Compared with some complex algorithms, the particle swarm-fish swarm algorithm is simpler and easier to implement. Because of its adaptive ability, it can adjust the search step according to the distribution network, and it is easier to adapt to the optimization demand of reducing network loss.

### Radial distribution network model building based on PSO and AF

The PSO workflow emulates the affine behavior of bird flocks during feeding. The search area for PSO is established as a forest within a regional territory, with particles representing the birds feeding within it. Then the bird feeding location is referred to as the global optimum, and the bird that discovers the food is recognized as the optimal solution, causing the remaining birds to gradually converge towards it. In the iterative process of PSO, the particle position update method is based on Eq. (1)^14^.

1 $$x_{i,d}^{t + 1} = x_{i,d}^{t} + v_{i,d}^{t + 1}$$

In Eq. (1), $d$ is the dimension of the forest in which the flock is working. The number of birds is given as $i$. $t$ represents the number of velocity updates. The position of the bird at this time is given as $x_{i,d}^{t}$. $v_{i,d}^{t + 1}$ represents the velocity update method of the particle, as shown in Eq. (2)^15^.

2 $$v_{i,d}^{t + 1} = \omega v_{i,d}^{t} + c_{1} r_{1} \left( {p_{i,d}^{t} - x_{i,d}^{t} } \right) + c_{2} r_{2} \left( {p_{i,d}^{t} - x_{i,d}^{t} } \right)$$

**Figure 1:**
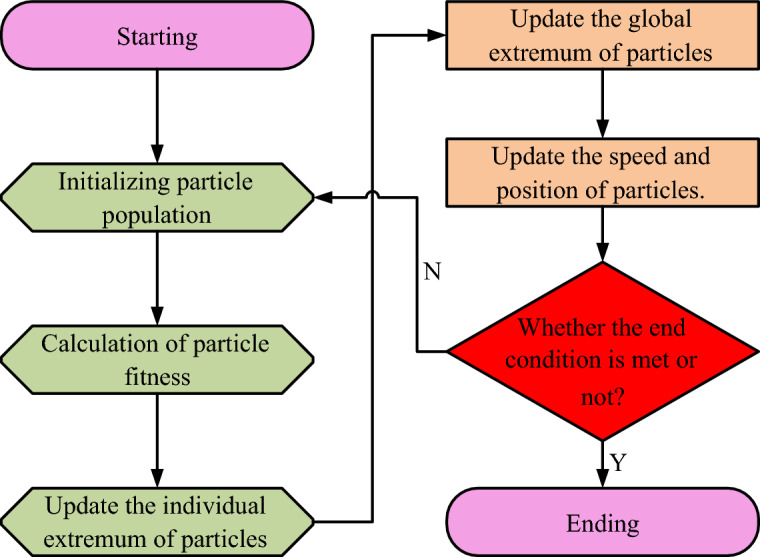
Working process diagram of PSO.

In Eq. (2), the speed of the bird at this time is denoted as $v_{i,d}^{t}$. The location of the food is called $p_{i,d}^{t}$. The learning medium of the flock is denoted by $c_{1} ,c_{2}$ and takes the value of 2. $r_{1} ,r_{2}$ takes the value on $\left[ {0,1} \right]$ and its value has to satisfy the randomness. The workflow diagram of PSO is shown in Fig. 1.

The complete PSO process includes particle initialization, particle adaptation and particle position update. The most important is the setting of various parameters. Too many PSO particles tend to fall into local optimum, and too few iterations tend to cause PSO to fail to find the optimal solution .The PSO particle velocity update method is used for mesh reconstruction, as shown in Eq. (3)^16^.

3 $$Sigmond\left( x \right) = \left( {1 + e^{ - x} } \right)^{ - 1}$$

When $v_{i,d}^{t} = x$, $Sigmond\left( {v_{i,d}^{t} } \right)$ gradually converges to $1$ as $v_{i,d}^{t}$ increases. As a mature algorithm, the PSO parameters are adjusted within a narrow range without impacting the end-result, and only require information consultation to configure them in a stepwise manner. During the operation of the PSO, there is sensitive parameter $\omega$, which is closely associated with the particle update rate of the PSO. When the value of $\omega$ is taken larger, the global search range of PSO is large. It facilitates the particles to jump out of the local optimum. When the value of $\omega$ is taken smaller, the working time of the algorithm is short. However, the improper value of $\omega$ will make the PSO's later curve oscillation interval larger. So Eq. (4) is established for the value of $\omega$ ^17^.

4 $$\omega = \omega_{\max } - \frac{{\left( {\omega_{\max } - \omega_{\min } } \right)t}}{T}$$

In Eq. (4), the maximum number of iterations of PSO is denoted as $T$. The number of iterations in which is denoted as $t$. The maximum and minimum values of $\omega$ are denoted as $\omega_{\max } ,\omega_{\min }$, respectively. Therefore, the objective function of the model can reduce the line loss in the power transmission system by changing the topological structure of the power grid. Because of the anti-interference ability of the power grid system, the fault distributed power supply can still maintain stable power supply. In addition, the objective function of distribution network reconfiguration model has constraints, which means that the obtained reconfiguration scheme meets various requirements of power network operation. In this study, the actual equipment configuration of the power grid is considered, and the topological structure of the power grid is maintained, so as to meet the arrangement mode between nodes. When the power supply load of each node reaches balanced distribution, the operation of distribution equipment is within the rated capacity range. The survival strategy of AF is demonstrated in its search for food. This means that the fish are attracted to areas with abundant food sources, while also enhancing AF's capacity to excel in its search, as demonstrated by Eq. (5)^18^.

5 $$h = E/\lambda T^{ - 1}$$

In Eq. (5), the survival difficulty of the fish population is denoted as $h$. The current energetic food position is represented by $E$. The iterative period of the population is denoted as $T$. $\lambda$ represents the energy lost by the fish population due to productive activities at the regional time. The competition mechanism of AF is reflected in the competition among individual fish, targeting the position with the maximum energy. The interspecific competition in AF not only promotes the strongest individuals to cross over competitively, but also enhances the local search ability of fish that have not reached the optimal position, as shown in Eq. (6)^19^.

6 $$E = \frac{{\varepsilon E_{\max } }}{\lambda }$$

**Figure 2:**
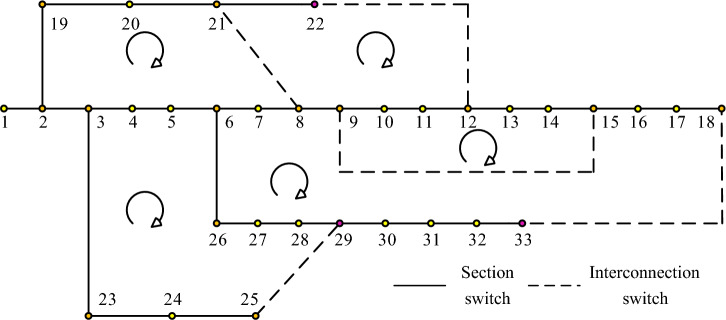
Two dimensional power network topology diagram.

In the above Eq. (6), the energy levels at the location of the globally optimal fish are denoted as $E_{\max }$ and the scale factor between them is expressed using $\varepsilon$. The motion characteristics of the particles are consistent with randomness. So the two-dimensional topological map of the complete grid is difficult to open the loop. To avoid this problem, a grid identification structure is set up that enables the calculation of the loss weights of the grid in the branches by observing the ring state of the smallest branch in the grid. Then the loop operation is reconstructed, as shown in Fig. 2.

In Fig. 2, it has a total of 33 grid stops, 32 segment switches and 5 contact switches. The use of circular arrows to indicate a closed loop helps to prevent local overvoltage at the nodes of the loop. The distribution network shown in Fig. 2 can be stepwise as solving the conductance matrix and impedance matrix when calculating the busbar. However, this method has some limitations when the number of network nodes is increased and the impedance range is increased. To reduce this effect, the improved equation proposed by Niura is shown in Eq. (7)^4^.

7 $$\Delta P/\Delta Q = \left[ {\begin{array}{*{20}c} H & N \\ J & L \\ \end{array} } \right] = \left[ {\begin{array}{*{20}c} {\Delta \delta } \\ {\Delta U/U} \\ \end{array} } \right]$$

In Eq. (7), $\Delta P$ represents the active power of the grid and the reactive power is represented by. $\Delta Q$,$H,N,J,L$ represent the voltages at the four critical points in the grid. The difference in potential between the initial and final points is noted as $\Delta U$. The phase voltages are represented by $\delta$, and $\cos \delta$ takes the value of $\left[ {0,0.5\pi } \right]$. The Oxla equation establishes the relationship between potential and electrical power, without taking into account the impact of resistance and reactance. To address this limitation, the study proposes the Oxla correction equation in Eq. (8) below^20^.

8 $$\left\{ {\begin{array}{*{20}c} {\Delta P/U = - B^{\prime}\Delta \theta } \\ {\Delta Q/U = - B^{\prime}\Delta U} \\ \end{array} } \right.$$

In the above Eq. (8), $B^{\prime}$ represents the error of the current accidentally doing work. The Oxla equation converges quickly in calculating the tidal current, but there are certain requirements for the first selected value. It is easy to fall into local optimum when the selected value is not reasonable.

### Improved PSO-AF model for distribution network reconfiguration

A common problem when applying the PSO-AF model to reconstruct distribution networks with low-voltage distributed power sources is falling into local optimum and narrow search objectives. To solve the early termination problem of the PSO algorithm, a binary code is added to PSO. It is used to improve the operational efficiency of the algorithm, as shown in Eq. (9) below^21^.

9 $$\left\{ {\begin{array}{*{20}c} {x_{i,d}^{t + 1} = 0} & {r_{i,d} = \min \left[ {r_{ij} } \right],ij \in K} \\ {x_{i,d}^{t + 1} = 1} & {otherwise} \\ \end{array} } \right.$$

In the above Eq. (9), $r_{i,d}$ represents the binary coding constant, which takes values in the range of $\left[ {0,1} \right]$. All the switching data sets in the loop circuit are noted as $K$. Using Eq. (9) to update the particle positions, even if the working circuit has a break position, it can be avoided by means of the loop to it, thus jumping out of the local optimum. The introduction of binary PSO increases its workload. To ensure the efficiency of the improved algorithm, the disconnected distribution of discrete branches is designed based on circuit loopback, as shown in Eq. (10)^22^.

10 $$\left\{ {\begin{array}{*{20}c} {\beta \left( {Yl} \right) = N^{ - 1} ,l = 1,2, \ldots ,N} \\ {\beta \left( {Zk} \right) = \frac{1}{M - 1},k = 1,2, \ldots ,\left( {M - 1} \right)} \\ \end{array} } \right.$$

In Eq. (10), the loop break probability is denoted as $\beta$. The total number of bridge-loop circuits is represented by $Y$. $l$ represents the bridge-loop of the current circuit. The number of screw-loops of the circuit is denoted by $Z$. The current screw-loop is denoted as $k$. The PSO dynamic model constructed using Eq. (10) correlates the network loss movement of the composite system. Assuming that the changes in the weight coefficients are 2, 1, and 2 in the regional time. Then the network loss changes during this time can be described by Eq. (11)^23^.

11 $$\min Fara = \sum\nolimits_{k = 1}^{3} {f_{k} \Delta t_{k} = 2f_{\max } } + f_{mid} + 2f_{\min }$$

**Figure 3:**
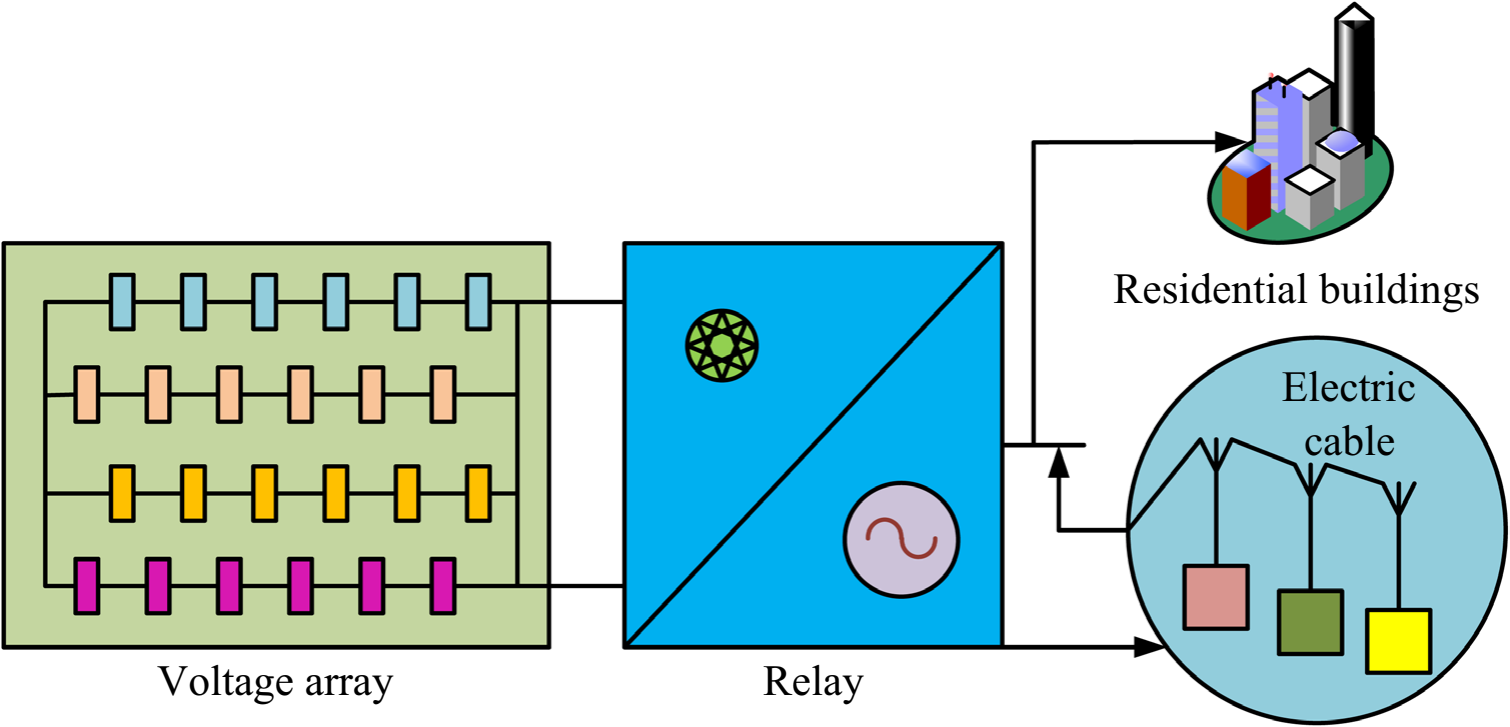
Schematic diagram of experimental power grid circuit.

In Eq. (11), the fluctuation range of network loss over a period of time is recorded as $Fara$. The influence of time in it is indicated by $\Delta t$. $f_{k}$ represents the net loss at the current time. The maximum, median, and minimum net loss for this time are denoted as $f_{\max } ,f_{mid} ,f_{\min }$. $k$ in which the value range is $k \in \left[ {0,\max } \right]$. The improved PSO by Eq. (11) enables a complete mapping of the net loss at the regional time, which allows the model to accurately feed back the life error. Life error refers to the process of circuit selection in distribution network, which has a negative impact on distributed power supply. It usually refers to an unexpected event or an already happened result. The conventional distribution network consists of voltage arrays, relays, closed grids and user groups. Based on this, an experimental circuit is constructed as shown in Fig. 3.

Figure 3 shows the circuit diagram for this experimental application. Since this distributed power supply consists of voltage modules connected in parallel, the effects caused by electrical heating can be maximized. The use of a double separation mode to build the relay also results in a shorter reactance length. To expand the search area of the AF, the multi-objective for the fish population in the AF is optimized, and then the optimal solution in the changing environment is found. For the stable power supply of the distributed power supply, the formula is established as shown in Eq. (12)^24^.

12 $$\min \phi_{1} = \sum\nolimits_{{\phi_{1} = 1}}^{n} {\frac{{\left( {V_{i} - V_{iN} } \right)^{2} }}{{V_{iN}^{2} }}}$$

In Eq. (12), the total number of branches of the distribution network is noted as $n$. The branch currently located is represented by $i$. $V_{i}$ represents the actual operating potential difference of that branch, and the rated voltage here is represented by $V_{iN}$. The optimized AF using Eq. (12) expands the fish population to some extent, but also takes into account the elite fish in the population. Elite fish have a larger search range and that gene should be better inherited. The elite fish have a requirement for the crowding of the shared radius, which is calculated as shown in Eq. (13)^25^.

13 $$s_{i} = \sum\nolimits_{j = 1}^{m} {\left( {\left| {\chi_{j}^{i + 1} - \chi_{j}^{i - 1} } \right|} \right)}$$

**Figure 4:**
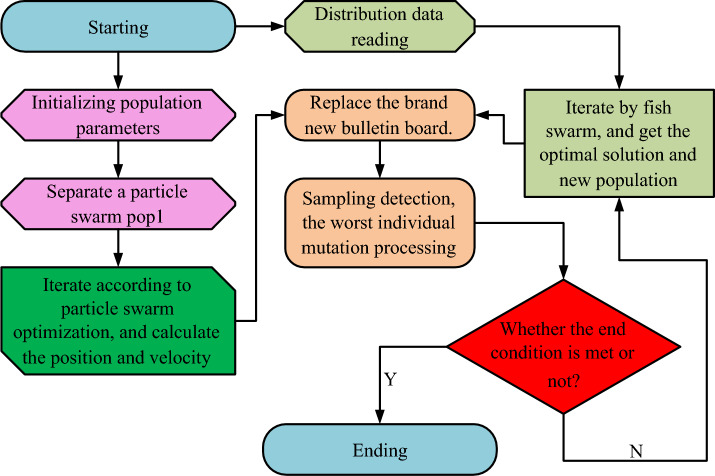
Workflow Diagram of Fusion Algorithm of bPSO-eAF.

In Eq. (13), the experimental extraction point is denoted as $i$. The crowding degree of this point is denoted by $s_{i}$. The total number of constraints is $m$. The number of targets is $j$, and the function of $j$ on $i$ is denoted as $\chi_{j}^{i}$. According to Eq. (13), the nearby crowding of the elite fish can be calculated to extract the optimal survival position of the fish. Then the superior individual in the offspring is selected and the two are merged. The operation is repeated continuously until the set number of times is reached. Finally fusing the PSO optimized by binary encoding with the AF considering elite fish generates the fusion algorithm bPSO-eAF. The flow is shown in Fig. 4.

The workflow of the bPSO-eAF algorithm shown in Fig. 4 can be divided into four steps. Through the complete flow of the algorithm, the optimal solution can be screened out from the distribution network data. The solution set that does not meet the requirements can be blended with the parents. When reconfiguring the network, it is studied to limit the traffic of specific lines through switches. These tools can be adjusted according to the real-time situation of network traffic to ensure that network line restrictions are maintained. For the parameters of network lines, the quality strategy is selected and the distributed parameters are adjusted according to the distribution network flow. Through the above method, the line restriction is maintained in the reconfigured network, and the line parameters of the used network are also selected. This study thinks that there are obstacles in the operation of POP2 process in this experiment, which easily makes the model fall into local optimum in the work, and then affects the experimental results of the research. In order to reduce this loss, the research thinks that the accuracy of the experiment is more important, so after comprehensive consideration, the research deletes the POP2 process in this experiment. During the operation of the distribution network, the power of the transformer is considered as the loss output. Equation (14) was established to calculate its losses^26^.

14 $$\left\{ {\begin{array}{*{20}c} {\iota_{0z} = \iota_{0} + K_{Q} Q_{0} } \\ {\iota_{kz} = \iota_{k} + K_{Q} Q_{k} } \\ \end{array} } \right.$$

In Eq. (14), the output power of the transformer as the only appliance is given by $\iota_{0z}$. Its power loss at rated resistance is expressed by $\iota_{kz}$. The coefficients when the circuit is not energized are given by $K_{Q}$. $0,k$ refer to the operating moment and the current operating cycle, respectively. Under ideal operating conditions, assuming the presence of multiple transformers, their fault (e.g. short circuit) times are calculated using Eq. (15)^27^.

15 $$\left\{ {\begin{array}{*{20}c} {u = \frac{{u_{1} u_{2} \left( {r_{1} + r_{2} } \right)}}{T}} \\ {t = ur} \\ {r = \frac{{r_{1} r_{2} }}{{r_{1} + r_{2} }}} \\ \end{array} } \right.$$

In Eq. (15), the operating time is recorded as $T$. The probability of failure at that time is expressed using $u_{1} ,u_{2}$. The time required to restore the operating condition is recorded as $r_{1} ,r_{2}$. So the failure time can be expressed using $u$. In the low-voltage circuit with a power supply of 35 kV, the overload inspection for each electrical appliance is studied to determine the loss of the circuit and determine the method of relay protection. After that, the risk of debugging each device in the circuit is studied first. The position of the device is determined to ensure that the faulty device is replaced in time. Then, the power supply is set to the normal line voltage, and the parameters of the device are debugged accordingly. Then according to the bus of the power supply, the maximum current in the circuit is estimated. The performance of the device is judged according to the overload current of the device. Finally, to prevent the reverse current in the circuit, the diode is put in the circuit ^28^. The pseudo-code formed by the model is shown in Table 1.

**Table 1:** Model pseudocode.

Initialize population of particles in b-PSO or fishes in e-AF
Initialize best position and fitness value for each particle or fish
Initialize global best position and fitness value
While (not reach stop criterion) do
For each particle or fish do
Update velocity based on b-PSO formula
Update position based on e-AF formula
Evaluate fitness value If (local best fitness value < current fitness value) then
Update local best position and fitness value
End If
If (global best fitness value < current fitness value) then Update global best position and fitness value
End If
End For
End While

## PSO-AF-based distribution network reconfiguration model experiments for low-voltage distributed power supplies

This chapter constructs a bPSO-eAF model based on low-voltage distributed power supplies to verify the effectiveness of the bPSO-eAF algorithm in real distribution networks with iterative, accuracy validation. Finally, the bPSO-eAF model is applied to conduct simulation experiments on the Elecgrid dataset.

### Development environment and parameter determination for the improved algorithm bPSO-eAF model

This research is selected from the Elecgrid data set on the standard IEEE test system and has multiple objective functions. In the process of establishing this data set, the research first collects the relevant data of power system, including load and energy consumption, and cleans these data to remove the wrong data. Then integrate the data from different sources, and finally standardize the data to keep the data format and unit consistent. Through the above steps, the Elecgrid data set was established and became the data set for this study. Due to the limited variety of data, the dataset was divided into a training set and a test set in a ratio of 4:6. The equipment and software used in the experiments are shown in Table 2 specifically.

**Table 2:** Experimental Parameters.

Device type	Operating parameters or software
Data set	Elecgrid
Rated voltage	25.84 kV
Number of branches	Article 37
Spiral ring circuit	Five rings
Bridge ring circuit	32 loops
Language	Easy Chinese
Loop impedance	0.2501 + j0.4249
Peak load	42 kW
Particle number	25
Network loss of closed circuit	202.4 kW
Model	Matlab R2018b
Computer chip	Intel i7 CPU

**Figure 5:**
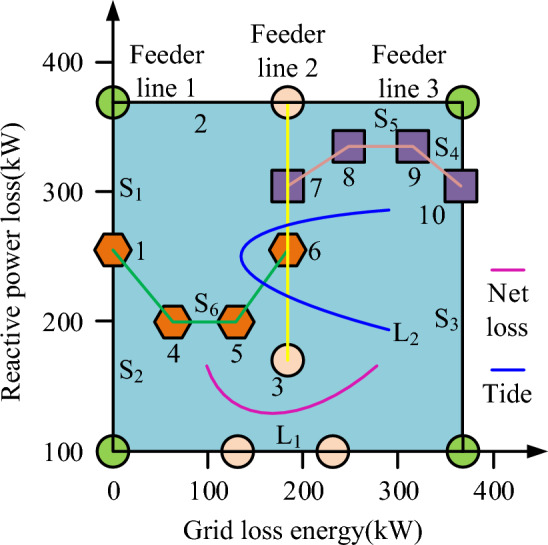
Power loss of electrical and net loss of lines when circuits are idling.

In a distribution network with distributed power sources, the objectives to be optimized usually consist of multiple ones. If the requirements of simultaneously correcting all objectives and running important objective functions are met, the optimal state compromise solution can be found among the dominant solutions. Based on the optimized objective function, dominant and non dominant experiments were established. The purpose is to calibrate the reactive power loss of each user and the network loss of the line when the circuit is idle. The calibration results are shown in Fig. 5. Figure 5 shows the experiments between the disposable and non-disposable objective functions in the three-feed line diagram. S_5_ represents the loss variation of the objective function to reach the optimal state. S_6_ represents the network loss of the disposable objective with the maximum load of 10. S_1_, S_2_, S_3_ and S_4_ represent the switches of the three feedback curves, respectively. By adjusting the on/off of the switches, the three feedback curves can be controlled, and then the idle state of each branch can be adjusted.

**Figure 6:**
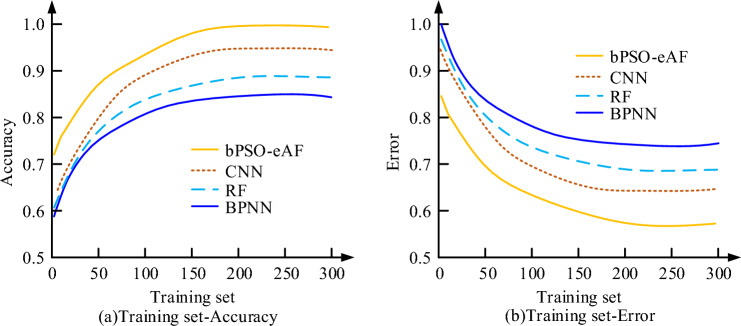
Four algorithm diagrams of accuracy and error rate in training set.

In Fig. 5, the reactive power loss and line network loss are 162 and 245 kW, respectively, under the condition of circuit no-load. This is because the distribution network reconfiguration models are mutually restricted, and they will change due to specific conditions in the actual distribution network reconfiguration, thus realizing the effective access of distributed power sources. The calibrated circuit requires further processing of the collected dataset before conducting experiments. The experiment utilizes bPSO-eAF for iterative optimization and compares Convolutional Neural Network (CNN), Random Forest (RF), Back Propagation Neural Network (BPNN), and fusion algorithms. During them, CNN can automatically learn features from original data and is widely used in various reconstruction technologies. It has translation invariance and local perception, and can extract the characteristics of power grid and reconstruct it through a series of layers when dealing with problems. RF is an integrated learning method, which performs classification and regression tasks by combining multiple decision trees. This method can reduce the over-fitting phenomenon through the integration of multiple decision trees, and then improve the accuracy and generalization ability of the model. And when dealing with data sets with a large number of features, RF needs feature selection or dimensionality reduction. Because there are a large number of missing values and abnormal values in the distribution network, this method has a strong pertinence for them. The method of BPNN is gradient descent, which has a good performance in classification and regression problems. It is composed of multiple neurons, and contains forward and reverse propagation paths, which can effectively screen the abnormal values of data. This process minimizes the loss function by updating the network parameters. These three methods can learn complex nonlinear relations, and also have good performance for a large number of features and samples, which has strong comparative significance in distribution network reconstruction. The training set accuracy and error rate results are displayed in Fig. 6.

**Figure 7:**
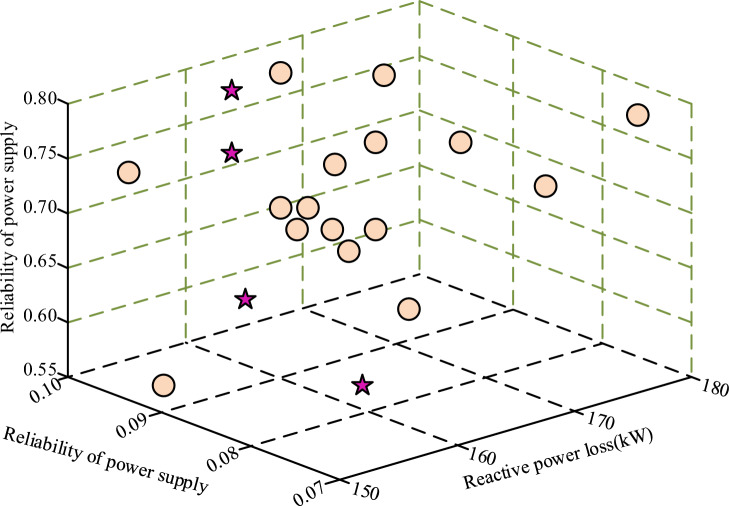
Changes of circuit load and power supply stability with reactive power loss.

In Fig. 6, the accuracy of the bPSO-eAF algorithm stabilizes at 155 iterations as the number of iterations increases. The accuracy rate is higher than the other three algorithms at this time, and the error rate is also at the lowest position. After 155 iterations, the increase of the accuracy and error rate of bPSO-eAF with the increase of the number of iterations are not significant. The impact on the experimental results is small and can be ignored. Taking into account the cost saving and the efficiency of the algorithm, the number of iterations was decided to be 155. The bPSO-eAF algorithm learning is finished, accounting for variations in load and power supply stability caused by rising reactive power loss, as depicted in Fig. 7.

In Fig. 7, with the increase of the circuit reactive power loss, the circuit load and power supply stability show irregular changes. So it is only possible to find a moment when three values can make the circuit present the best working condition. When the reactive power loss value of the circuit is exactly 162 kW, the stability value of the power supply is 0.083. It is sufficient to support the needs of the experiment. The circuit load is 0.72, which can afford the operation of the fixed-value resistor.

### Experimental verification of reconfigured distribution network with bPSO-eAF at 25.84 kV power supply

**Figure 8:**
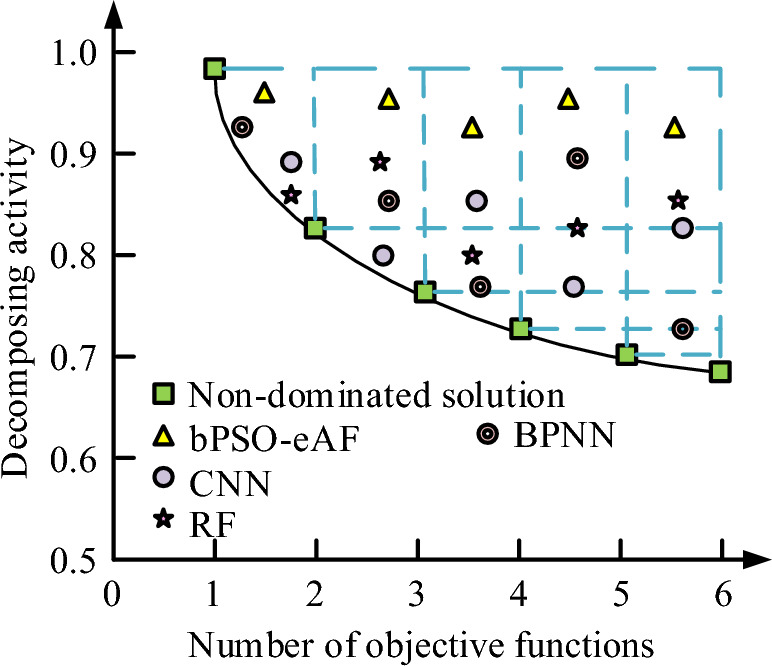
Illustration of research on activity of disposable solution of four algorithms.

In a simulation experiment to validate the bPSO-eAF algorithm model, the parameters are set, in a circuit consisting of at most six objective functions. The liveliness of the disposable solution sets of the four algorithms is explored for the case where the number of infeasible solution sets is rated. Different graphs are used to represent the various algorithms. The solutions with activity values beyond the infeasible solution sets are set as the dominant solution sets. The number of objective functions is used to represent the disposable solution set activity of the four algorithms under the condition that the number of infeasible solution sets is rated. The experimental results are plotted as shown in Fig. 8.

**Figure 9:**
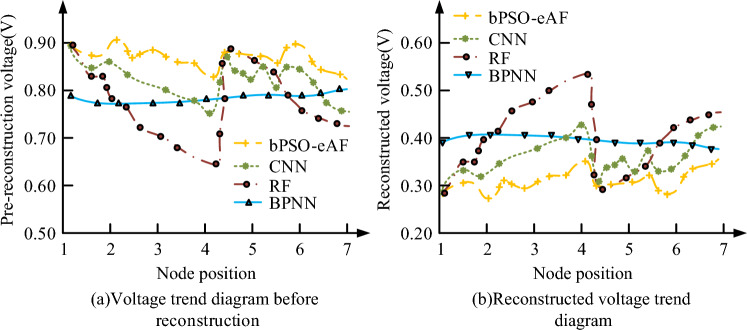
The potential difference changes of the four algorithms during reconstruction.

As Fig. 8, when the circuit is composed of 5 objective functions, bPSO-eAF has a dominant solution set activity of 0.94, which is close to 1 and outperforms the other three algorithms. The activity of bPSO-eAF is lower when the objective functions are 2, 3, 4 and 6 compositions, but still higher than CNN, RF and BPNN. Before and after reconfiguring the circuit, the voltage in the circuit changes. Therefore, the potential difference between the four algorithms is analyzed during the reconfiguration process, and the results are presented in Fig. 9. Distribution network reconfiguration can plan and design the distribution network according to the existing load demand, so as to determine the reasonable network structure. He can upgrade and replace the backward equipment. This includes replacing old transformers and protective devices, and installing new intelligent equipment, thus improving the reliability and operating efficiency of the equipment. He can intelligently introduce automation technology, including installing smart meters and remote monitoring systems to realize real-time monitoring of distribution networks.

**Figure 10:**
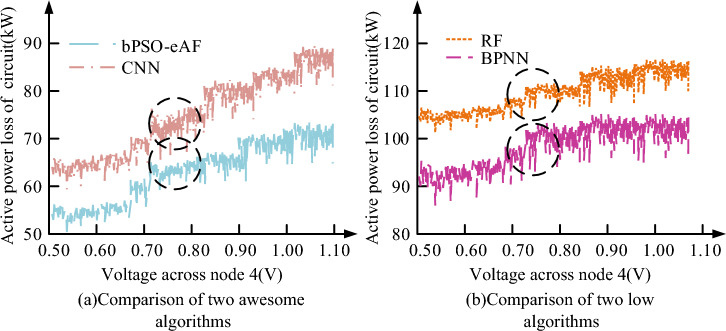
Active power loss of the disposable solution set on node 4.

In Fig. 9, the voltage value of bPSO-eAF at node 4 before reconstruction is the highest, and the voltage values of bPSO-eAF, CNN, RF, and BPNN here are 0.83, 0.76, 0.64, and 0.77 V, respectively. The voltage value of bPSO-eAF at node 4 after reconstruction is the lowest, and the voltage values of the four algorithms at this time are 0.29, 0.32, 0.31, and 0.39 V, respectively. The optimization effect of this operation for bPSO-eAF is stronger than the other three algorithms, and the reconfigured bPSO-eAF algorithm works optimally. Therefore, to analyze the power loss of the prominent solution set in an active manner, the potential difference variation is measured at both ends in the presence of the consumer. The image is illustrated in Fig. 10.

**Figure 11:**
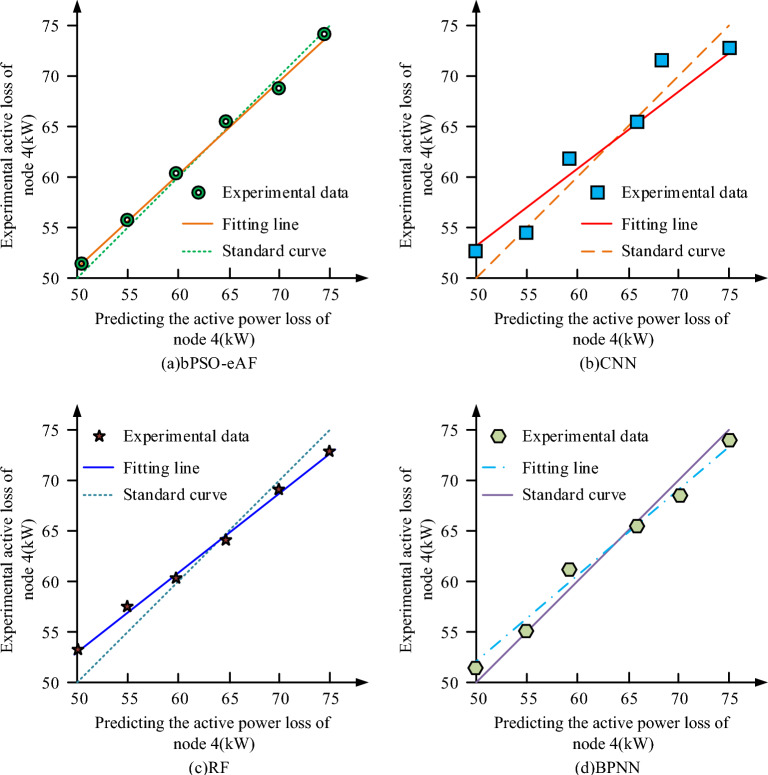
Extensive experiments of four algorithms.

As the voltage increases, all the algorithms in Fig. 10 do more extra work. In Fig. 10, Awesome algorithms means that they perform better in distribution network reconfiguration, while low algorithms means that their performance in distribution network reconfiguration experiment is not satisfactory. Since the device on node 4 operates at 0.74 V, the study only analyzes the active power losses of the four algorithms at 0.74 V. From Fig. 10, the bPSO-eAF algorithm has the lowest active power loss value of 63 kW. As Fig. 10, the bPSO-eAF algorithm has the lowest loss value of 63 kW. The active power loss values of CNN, BPNN and RF are 74, 97 and 109 kW, respectively. However, the results obtained from one experiment cannot meet the requirement of algorithm extensiveness. So six experiments are conducted under the same conditions and the results of each experiment with the expected value are compared. The linear fit image is ploted as shown in Fig. 11. Due to the rapid development of new energy sources, distribution network reconstruction needs to consider distributed energy sources, such as solar photovoltaic systems. Among them, adjusting the structure of distribution network accounts for a large proportion, which can realize the smooth access of sustainable energy. This paper studies how to strengthen the safety and reliability management of distribution network by improving the automation system of equipment. It can increase the redundancy of equipment, and then improve the fault detection ability. The data show that distribution network reconfiguration is a complex comprehensive project, and it should take into account many factors such as technology, economy, environment and society. Study and use reasonable planning and design to improve the reliability of power supply. Under the condition of increasing load demand, this method can promote energy transformation and sustainable development. At the same time, the calculation delay research experiment of the algorithm is studied, and the experimental results of the calculation delay research show that the calculation delay parameters of the algorithm perform well.

Figure 11 shows the comparison between the predicted active loss and the true active loss for the four algorithms at node 4. From Fig. 11, the linear fit of the bPSO-eAF algorithm ($R^{2}$) is 0.9804, while the $R^{2}$ of CNN, RF and BPNN are 0.9527, 0.9612 and 0.9503, respectively. This indicates that none of the four models has underfitting. In summary, it can be concluded that the bPSO-eAF algorithm model has higher accuracy and lower loss, and is suitable for distribution network reconfiguration under low voltage power supply.

## Results and discussion

The motion characteristics of electrons conform to randomness, so it is difficult to open the two-dimensional topology diagram of a complete power grid. This paper studies the setting of power network identification structure in distribution network, and improves the voltage distribution in the process of voltage transportation by observing the ring state of the smallest branch in the power network. The goal of this study is to improve the particle swarm-fish swarm fusion algorithm and apply it to the distribution network reconfiguration of distributed generation at 35 kV. The bPSO-eAF algorithm is proposed and used to optimize the structure and operation mode of distribution network to improve the reliability of the system. Through experiments and simulations, the results are obtained and discussed.

This study successfully reconstructed the distribution network, and the reconstructed distribution network has better performance and can quickly respond to power demand and faults. This is very similar to the results obtained by Yang et al.^29^. In the research of system reliability, it is studied that the probability of power failure is the lowest among the four algorithms by reasonably configuring the line connection mode, which significantly improves the fault tolerance of the system. Hu et al.^30^ obtained similar results. For the economic research of the system, the energy consumption and operating cost of the system are lower than those of CNN, RF and BPNN systems. The study also improves the efficiency of power supply, so that the reconstructed distribution network can improve the sustainability of the system. In addition, the study also studies the optimization of energy scheduling strategy, and effectively reduces the dependence on traditional energy sources, thereby reducing carbon emissions.

In a word, the bPSO-eAF algorithm proposed in this study has made pioneering progress in the research of distribution network reconfiguration of distributed power sources below 35 kV. Studying and optimizing the structure and operation mode of distribution network can improve the reliability, economy and sustainability of the system and provide useful reference for future power supply. However, the related technical management problems faced by the research still need further practice to solve. In addition, there are still some technical and management problems in the access and management of distributed power sources, power load forecasting and dispatching. These problems will be solved step by step in future research.

## Conclusion

Under the background of high-speed development of distribution network in low-voltage distributed power supply industry, research on energy loss reduction for long-distance power transmission is becoming increasingly important. This paper generates fusion algorithm (bPSO-eAF) based on variant bird and elite fish optimized PSO-AF. The efficiency of power supply and relay is also considered. The data from Elecgrid’s self-built dataset is used for distribution network reconstruction experiments and compared with CNN, RF, and BPNN. Through the testing of various experimental parameters, the stability value of the power supply is determined to be 0.083, the circuit load is 0.72, and the number of iterations is set to 155. When the circuit consists of five objective functions, the bPSO-eAF can govern the solution set activity of 0.94, which is the highest activity among the four algorithms. The voltage values of the four algorithms after reconstruction are 0.29, 0.32, 0.31 and 0.39 V, which shows that the reconfigured bPSO-eAF algorithm works best. The bPSO-eAF algorithm has the lowest active loss value of 63 kW when using 0.74 V. The active loss values of CNN, BPNN and RF are 74, 97 and 109 kW, respectively. bPSO-eAF has the lowest loss in the results of six experiments in the extensive tests. The experimental results show that the bPSO-eAF algorithm can significantly reduce the active losses in distribution network reconfiguration. It is found that this method is suitable for long-distance distribution network reconfiguration under low-voltage power supply. The actual achievement of the research is to effectively reduce the resistance of long circuits, thus reducing the active power loss in the process of power transmission. However, the experiment is set to a low voltage environment below 35 kV, and the influence of high voltage on bPSO-eAF is ignored. When the voltage at both ends of the circuit is set to high voltage, bPSO-eAF model will isolate it into isolated points when dealing with distribution network reconfiguration. This will cause great errors to the experimental results. Moreover, the protective measures of the circuit are rubber protective sleeve, and the insulation layer will be broken down when the voltage is higher than 35 kV, thus endangering the personal safety of the experimenter. This will be the next step in the research as protection measures are developed.

## Data Availability

The datasets used and/or analyzed during the current study are available from the corresponding author on reasonable request.
